# Cigarette Smoking Among US Adults With Selected Chronic Diseases Associated With Smoking, 2010–2019

**DOI:** 10.5888/pcd19.220086

**Published:** 2022-09-29

**Authors:** Caitlin G. Loretan, Monica E. Cornelius, Ahmed Jamal, Yiling J. Cheng, David M. Homa

**Affiliations:** 1 Office on Smoking and Health, National Center for Chronic Disease Prevention and Health Promotion, Centers for Disease Control and Prevention, Atlanta, Georgia

## Abstract

**Introduction:**

People who smoke cigarettes are at greater risk of developing chronic diseases and related complications. Our study provides recent estimates and trends in cigarette smoking among people with respiratory and cardiovascular diseases, cancers, and diabetes.

**Methods:**

Using data from the 2019 National Health Interview Survey, we calculated the prevalence of current and former cigarette smoking among adults aged 18 to 44 years, 45 to 64 years, and 65 years or older with chronic diseases. Those diseases were cancers associated with smoking, chronic obstructive pulmonary disease, diabetes, coronary heart disease, and/or stroke (N = 3,741). Using data from the 2010–2019 National Health Interview Surveys, we assessed trends in current cigarette smoking by chronic disease by using the National Cancer Institute’s Joinpoint Regression Program.

**Results:**

In 2019, current cigarette smoking prevalence among adults with chronic diseases associated with smoking ranged from 6.0% among adults aged 65 or older with diabetes to 51.9% among adults aged 18 to 44 years with 2 or more chronic diseases. During 2010 through 2019, a significant decrease occurred in current cigarette smoking among adults aged 45 to 64 years with diabetes.

**Conclusion:**

Overall, smoking prevalence remains high and relatively unchanged among people with chronic diseases associated with smoking, even as the overall prevalence of cigarette smoking in the US continues to decrease. The lack of progress in smoking cessation among adults with chronic diseases associated with smoking suggests that access, promotion, and integration of cessation treatment across the continuum of health care (ie, oncology, pulmonology, and cardiology settings) may be important in the success of smoking cessation in this population.

**Summary**

**What is already known on this topic?**

Cigarette smoking has been linked to more than 27 diseases, including respiratory and cardiovascular diseases, cancers, and diabetes.

**What is added by this report?**

In 2019, more than 1 in 4 US adults aged 18 to 64 years with at least 1 chronic disease associated with smoking reported that they currently smoke. During 2010 through 2019, the only significant decrease in cigarette smoking was found among adults aged 65 years or older living with diabetes.

**What are the implications for public health practice?**

The findings of this report may help identify groups of people who continue to smoke and could potentially benefit from access, promotion, and integration of cessation treatment across the continuum of health care.

## Introduction

Chronic diseases associated with cigarette smoking include respiratory and cardiovascular diseases, cancers, and diabetes (1). An estimated 16 million US adults live with a smoking-related disease (1). Cigarette smoking can increase the risk of chronic disease and subsequent complications and can lead to overall reduced quality of life (1). As of 2019, 34.1 million adults (14.0%) in the US currently smoke cigarettes (2).

Cigarette smoking is the predominant cause of lung cancer and chronic obstructive pulmonary disease (COPD). Furthermore, smoking increases one’s risk of cardiovascular disease, several types of cancer, diabetes, and other chronic conditions (1,3–6). Although many studies have evaluated the effect of smoking on chronic disease development, few studies have assessed the prevalence of current cigarette smoking among adults with chronic diseases. The most recent published estimates of cigarette smoking among adults with asthma, diabetes, heart disease, hypertension, hepatitis, HIV, lung cancer, or stroke were reported using data from the 2013 National Survey on Drug Use and Health (7).

The objectives of our study were to 1) provide the most recent (2019) estimates of current and former cigarette smoking among adults aged 18 years or older with chronic diseases that can be associated with smoking (hereinafter, chronic disease) and 2) report temporal changes in current cigarette smoking among adults with chronic disease during 2010 through 2019.

## Methods

### Study sample

We obtained data from the 2010–2019 National Health Interview Surveys (NHIS) to examine self-reported cigarette smoking behaviors among adults aged 18 years or older with chronic disease. We chose to include adults aged 18 years or older on the basis of prior research related to the prevalence of multiple chronic diseases (8). The NHIS is an annual, nationally representative, cross-sectional, household survey of the noninstitutionalized US civilian population that has previously been described in detail (9). In 2019, NHIS underwent changes to nonresponse survey weighting methodology and questionnaire redesign (10,11). Respondents with unreported age and missing cigarette smoking status were excluded (n = 878). Our analyses were conducted during 2020 through 2022. During 2010 through 2019, survey response rates for sample adults aged 18 years or older ranged from 53.0% in 2017 to 63.3% in 2011 (9,12).

### Measures

Current cigarette smoking was defined as a person having smoked 100 or more cigarettes in their lifetime and smoking every day or some days at the time of interview (2). Former cigarette smoking was defined as a person having smoked 100 or more cigarettes in their lifetime and not smoking at all at the time of interview.

### Chronic disease

Chronic diseases were assessed by self-report, asking participants if they had ever been diagnosed with any 1 of the 5 selected chronic diseases, or 2 or more. Chronic diseases were cancer (bladder, cervix, colorectal, esophagus, kidney, larynx, liver, lung, oropharynx, pancreas, stomach, trachea); COPD (emphysema, chronic bronchitis); diabetes; coronary heart disease (CHD); and stroke (1). Participants were included in the analysis as having a chronic disease if they answered yes to “Have you ever been told by a doctor or other health professional that you had [disease]?”, apart from CHD and COPD.

### CHD and myocardial infarction (MI)

Separate questions were asked for CHD or heart attack and MI, an outcome of CHD. Respondents were coded as having CHD if they answered yes to having been told they had CHD or if they answered yes to having been told they had a MI, regardless of their response for CHD.

### COPD

For COPD, participants were considered to have COPD if they answered yes when asked if they had ever been told by a doctor or other health professional that they had chronic obstructive pulmonary disease, also called COPD, 2) if they have ever been told by a doctor or other health professional that they had emphysema, or 3) if during the past 12 months they were told by a doctor or other health professional that they had chronic bronchitis.

### Two or more chronic diseases

Participants were included as having 2 or more chronic diseases if they reported more than 1 of the aforementioned chronic diseases assessed in this study. Disease categories were not mutually exclusive.

NHIS questions did not allow us to distinguish between type 1 and type 2 diabetes, although cigarette smoking increases the risk of developing type 2 diabetes (1). Data on kidney cancer was not accessible for 2019. Our analysis excluded acute myeloid leukemia because NHIS does not differentiate between leukemia and acute myeloid leukemia.

### Statistical analysis

We used 2019 NHIS data to calculate prevalence estimates and 95% CIs for current and former cigarette smoking among adults with chronic disease. We reported the prevalence of cigarette smoking by chronic disease for the following age groups: 18 to 44 years (young), 45 to 64 years (middle-aged), and 65 or older (older) (8).

We calculated the annual percentage change (APC) of current cigarette smoking from 2010 to 2019 for each chronic disease by age group using the National Cancer Institute’s Joinpoint Regression Program version 4.8.01 (SEER*Stat), which uses log-linear models and a Monte Carlo permutation test for significant changes in trend (13). Identification of a joinpoint at a given year indicates a significant change in trend. In the absence of joinpoints, APCs were considered constant and equal to average APC, which is a summary measure of APCs over a period of time (14). Significance was defined as *P* < .05 for trends. To calculate prevalence estimates, we first used variance estimation variables to account for the multistage complex sampling design of the survey. Data were then weighted to provide nationally representative estimates. In accordance with the 2017 National Center for Health Statistics guidelines, statistically unreliable estimates were suppressed (15). Analyses were conducted using SAS-callable SUDAAN software version 11.0.3 (RTI International).

## Results

In 2019, the unweighted NHIS sample contained 31,997 adults; of these, 3,741 (11.7%) reported current or former cigarette smoking and any chronic disease. Of these 3,741 participants, 262 (7.0%) were aged 18 to 44 years, 1,305 (34.9%) were aged 45 to 64 years, and 2,174 (58.1%) were aged 65 years or older.

Current cigarette smoking prevalence among young adults in the study ranged from 22.6% (95% CI, 16.4%–28.8%) among those with diabetes to 51.9% (95% CI, 37.4%–66.5%) among those with 2 or more chronic diseases (Table 1). Among the middle-aged group, current cigarette smoking prevalence ranged from 17.3% (95% CI, 15.0%–19.7%) among those with diabetes to 49.1% (95% CI, 44.2%–53.9%) among those with COPD. Among the older age group, current cigarette smoking prevalence ranged from 6.0% (95% CI, 4.7%–7.4%) among those with diabetes to 21.5% (95% CI, 18.4%–24.5%) among those with COPD.

**Table 1:** Prevalence of Cigarette Smoking by Chronic Disease Associated With Smoking Among Adults Aged ≥18 Years, National Health Interview Survey, US, 2019

Chronic disease, by age group	Current cigarette smoking,^a^ % (95% CI)	Former cigarette smoking, % (95% CI)
**Aged 18–44 y**		
Any chronic disease^b^	27.8 (23.3–32.3)	13.6 (10.6–16.6)
Chronic obstructive pulmonary disease	34.5 (25.5–43.4)	18.8 (11.5–26.2)
Coronary heart disease	34.4 (22.2–46.7	NR
Stroke	35.0 (23.1–46.8)	NR
Diabetes	22.6 (16.4–28.8)	14.5 (10.1–19.0)
Cancer associated with smoking^c^	45.3 (31.8–58.8)	NR
≥2 Chronic diseases	51.9 (37.4–66.5)	20.0 (9.0–31.0)
**Aged 45–64 y**		
Any chronic disease^b^	26.0 (23.9–28.1)	28.2 (25.9–30.5)
Chronic obstructive pulmonary disease	49.1 (44.2–53.9)	32.5 (28.2–36.9)
Coronary heart disease	26.6 (22.5–30.6)	32.0 (27.6–36.4)
Stroke	29.8 (22.8–36.8)	24.7 (19.3–30.2)
Diabetes	17.3 (15.0–19.7)	27.9 (25.0–30.8)
Cancer associated with smoking^c^	30.0 (22.5–37.4)	34.2 (26.0–42.4)
≥2 Chronic diseases	32.5 (27.8–37.1)	32.1 (27.6–36.7)
**Aged ≥65 y**		
Any chronic disease^b^	10.1 (9.0–11.3)	45.9 (44.0–47.7)
Chronic obstructive pulmonary disease	21.5 (18.4–24.5)	57.6 (54.1–61.1)
Coronary heart disease	7.8 (6.1–9.5)	51.3 (48.4–54.3)
Stroke	10.8 (7.8–13.7)	46.6 (42.5–50.7)
Diabetes	6.0 (4.7–7.4)	42.8 (39.9–45.8)
Cancer associated with smoking^c^	14.0 (10.1–17.9)	48.5 (43.1–53.8)
≥2 Chronic diseases	10.7 (8.6–12.9)	53.9 (50.4–57.3)

Abbreviation: NR, not reported.

a Currently, daily, or some days of cigarette smoking at the time of survey.

b Chronic disease includes any of the following associated with smoking: chronic obstructive pulmonary disease, coronary heart disease, stroke, diabetes, and cancer associated with smoking.

c Bladder, cervical, colorectal, esophageal, kidney, larynx or trachea, liver, lung, oropharynx, pancreas, and stomach cancers. Data for kidney cancer were not accessible for 2019.

Among young adults, the prevalence of former cigarette smoking ranged from 13.6% (95% CI, 10.6%–16.6%) among those with any chronic disease to 20.0% (95% CI, 9.0%–31.0%) among those with 2 or more chronic diseases (Table 1). Among middle-aged adults, the prevalence of former cigarette smoking ranged from 24.7% (95% CI, 19.3%–30.2%) among those with a history of stroke to 34.2% (95% CI, 26.0%–42.4%) among those with history of cancer. Among older adults, prevalence ranged from 42.8% (95% CI, 39.9%–45.8%) among those with diabetes to 57.6% (95% CI, 54.1%–61.1%) among those with COPD.

During 2010 through 2019, adults aged 45 to 64 years and those older than 65 years with COPD consistently had a high prevalence of current cigarette smoking (Figure). Among older adults with CHD, the prevalence of current cigarette smoking significantly increased during 2010 to 2016, then began trending downward. Additionally, among older adults with 2 or more chronic diseases, the trend in the prevalence of current cigarette smoking increased during 2010 to 2016, then decreased (Table 2). We did not find other joinpoints; therefore, all other APCs were considered constant and equal to average APC. We found a significant decrease in current cigarette smoking among middle-aged adults with diabetes.

**Figure:**
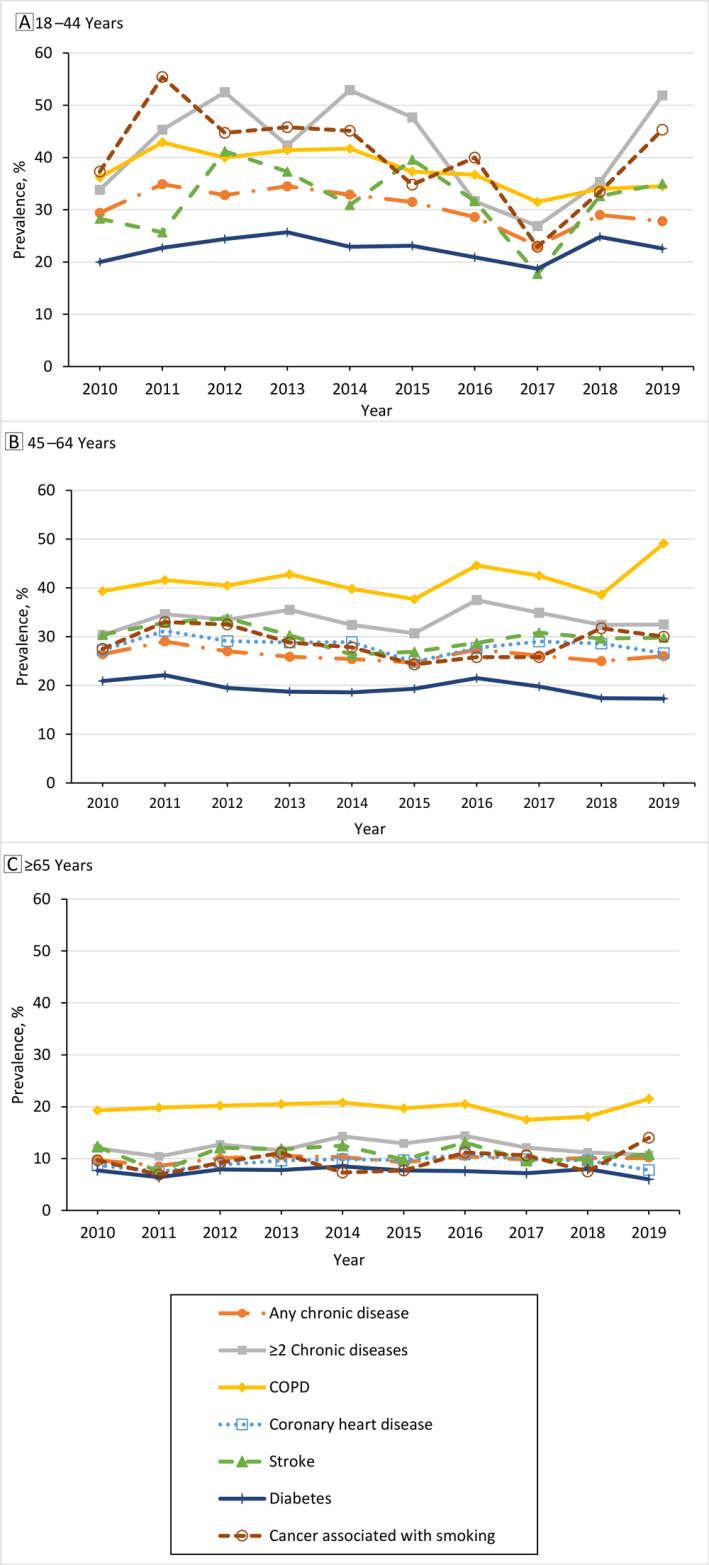
Trends in prevalence of current cigarette smoking by age group and chronic disease associated with cigarette smoking, National Health Interview Survey, 2010–2019. A, Participants aged 18 to 44 years. B, Participants aged 45 to 64 years. C, Participants aged 65 years or older. Abbreviation: NR, not reported.

**Table:** 

Year	Any chronic disease	≥2 Chronic diseases	Chronic obstructive pulmonary disease	Coronary heart disease	Stroke	Diabetes	Cancer associated with smoking
**Aged 18 to 44 years**							
2010	29.4	33.8	36.2	NR	28.3	20.0	37.3
2011	34.9	45.3	42.9	NR	25.7	22.7	55.4
2012	32.8	52.5	40.0	NR	41.2	24.4	44.7
2013	34.5	42.3	41.4	NR	37.3	25.7	45.8
2014	32.9	52.9	41.7	NR	30.9	22.9	45.1
2015	31.5	47.7	37.3	NR	39.6	23.1	34.8
2016	28.6	31.6	36.7	NR	31.7	20.9	40.0
2017	23.1	26.9	31.5	NR	17.7	18.7	22.9
2018	29.0	35.3	34.0	NR	32.6	24.8	33.4
2019	27.8	51.9	34.5	NR	35.0	22.6	45.3
**Aged 45 to 64 years**							
2010	26.4	30.3	39.3	27.2	30.4	20.9	27.5
2011	29.0	34.6	41.6	31.2	32.9	22.1	33.0
2012	27.0	33.5	40.5	29.1	33.8	19.5	32.5
2013	25.9	35.5	42.8	28.8	30.3	18.7	28.8
2014	25.4	32.4	39.8	28.9	26.3	18.6	27.8
2015	24.7	30.7	37.7	24.8	26.9	19.3	24.3
2016	27.4	37.5	44.6	27.7	28.7	21.5	25.8
2017	26.1	34.9	42.5	29.0	30.8	19.8	25.8
2018	25.0	32.4	38.6	28.6	29.6	17.4	31.7
2019	26.0	32.5	49.1	26.6	29.8	17.3	30.0
**Aged 65 years or older**							
2010	9.7	12.0	19.3	8.7	12.3	7.7	9.7
2011	8.6	10.4	19.8	7.5	7.5	6.4	6.9
2012	10.1	12.7	20.2	8.8	12.1	7.9	9.2
2013	10.5	11.6	20.5	9.6	11.8	7.8	11.1
2014	10.3	14.3	20.8	9.9	12.5	8.5	7.3
2015	9.3	12.9	19.7	9.7	9.8	7.7	7.7
2016	10.4	14.4	20.5	10.8	13.1	7.6	11.2
2017	9.8	12.1	17.5	9.9	9.6	7.2	10.6
2018	10.1	11.2	18.1	9.6	9.9	8.0	7.5
2019	10.1	10.7	21.5	7.8	10.8	6.0	14.0

**Table 2:** Trends in Current Cigarette Smoking by Age Group and Chronic Disease in the US, National Health Interview Survey, 2010–2019

Chronic disease, by age group	Current cigarette smoking^a^	
	Annual percentage change^b^ (95% CI)	*P* value
**Aged 18–44 y**		
Any chronic disease^c^	−2.3 (−4.8 to 0.3)	.07
Chronic obstructive pulmonary disease	−2.1 (−4.3 to 0.1)	.06
Coronary heart disease	NR	NR
Stroke	0 (−5.6 to 5.8)	.99
Diabetes	0 (−2.6 to 2.7)	.99
Cancer associated with smoking^d^	−3.5 (−8.6 to 1.9)	.17
≥2 chronic diseases	−0.9 (−6.5 to 4.9)	.71
**Aged 45–64 y**		
Any chronic disease^c^	−0.8 (−1.9 to 0.3)	.15
Chronic obstructive pulmonary disease	1.2 (−0.7 to 3.2)	.17
Coronary heart disease	−0.8 (−2.2 to 0.7)	.28
Stroke	−1.1 (−3.1 to 1.0)	.27
Diabetes	−1.8 (−3.4 to −0.1)	.04
Cancer associated with smoking^d^	−0.8 (−3.4 to 1.9)	.51
≥2 chronic diseases	0.3 (−1.5 to 2.1)	.75
**Aged ≥65 y**		
Any chronic disease^c^	0.7 (−0.7 to 2.2)	.28
Chronic obstructive pulmonary disease	−0.2 (−2.0 to 1.5)	.76
Coronary heart disease		
2010–2016	5.3 (0.3 to 10.5)	.04
2016–2019	−8.6 (−20.2 to 4.7)	.15
Stroke	−0.3 (−4.6 to 4.3)	.89
Diabetes	−0.8 (−3.5 to 2.1)	.54
Cancer associated with smoking^d^	3.8 (−1.6 to 9.6)	.15
≥2 Chronic diseases		
2010–2016	3.8 (−1.8 to 9.8)	.15
2016–2019	−9.6 (−21.9 to 4.7)	.07

Abbreviation: NR, not reported.

a Current, daily, or some days cigarette smoking at the time of survey.

b Comparison with 2019 must be interpreted with caution because of changes to nonresponse survey weighting methodology and a questionnaire redesign in 2019.

c Any chronic disease includes people with any of the following chronic diseases associated with smoking: chronic obstructive pulmonary disease, coronary heart disease, stroke, diabetes, and cancer associated with smoking.

d Bladder, cervical, colorectal, esophageal, kidney, larynx/trachea, liver, lung, oropharynx, pancreas, and stomach cancers; data for kidney cancer were not accessible for 2019.

## Discussion

This study found that cigarette smoking persists among adults with chronic disease. Cigarette smoking is most prevalent among young and middle-aged adults and among adults with a history of COPD and 2 or more chronic diseases. Cigarette smoking prevalence among middle-aged adults with diabetes decreased during 2010 through 2019.

In 2019, among adults with COPD, at least 1 in 5 participants reported current cigarette smoking. Several characteristics might make it harder for people with COPD to quit smoking. Some studies have found that people with COPD who continue to smoke may have greater nicotine dependency and smoke more cigarettes per day; inhale a greater volume of smoke, allowing for increased amounts of substances into the lungs; or might not have the self-esteem and motivation to eventually achieve smoking cessation (16,17). More than 1 in 3 young adults and almost 1 in 2 middle-aged adults with COPD reported cigarette smoking. This is an important finding because smoking cessation is the only established intervention that reduces loss of lung function among people with COPD, and the sooner a person quits smoking, the slower the rate of decline in lung function (18). These results are comparable to previous findings and are not surprising given that smoking is the dominant cause of COPD (16,19).

In our study we found that more than 1 in 4 adults aged 45 to 64 years with CHD currently smoked cigarettes. We did not find any significant temporal change in current cigarette smoking among adults with CHD. The association between smoking and cardiovascular disease is well established, with even low levels of cigarette exposure implicated in acute cardiovascular events, such as MI (1). A study reported that 52.5% of patients (median age 45 years) hospitalized with acute MI were currently smoking cigarettes, and 62.0% of those who smoked at the time of their MI continued to smoke after the event (20). Another study showed that adults who experienced a recent MI increased perception of the harm of smoking continuation and were more likely to report that they were attempting to reduce their smoking consumption or quit (21). However, there was no association between recent MI and smoking cessation (21). Results from earlier research using data from 2005 to 2013 reported increased prevalence of cigarette smoking among adults with heart disease and hypertension compared to adults without chronic disease (7). Additionally, a study using a large US registry found that only 1 in 3 adults who smoke cigarettes and were seen for a cardiology visit received smoking cessation services (22).

The prevalence of cigarette smoking among survivors of cancers associated with smoking reported in this study is higher than NHIS-based estimates of cigarette smoking among all cancer survivors reported by the National Cancer Institute (23). One possible explanation for our higher estimates is our restriction of cancer types to those that are causally associated with cigarette smoking. Similar to our findings, the National Cancer Institute reported a decrease in cigarette smoking with increasing age among all cancer survivors (23). Regardless of age and cancer type, it is important for all cancer survivors to quit smoking, because evidence suggests that smoking cessation has the potential to decrease all-cause mortality among all cancer survivors (18).

Current cigarette smoking can complicate treatment of diabetes and lead to increased risk of cardiovascular disease, kidney disease, reduced circulation, and loss of sight (1). Notably, we found a significant decrease in cigarette smoking among middle-aged adults with diabetes over time. A 2015 study reported that among people with type 2 diabetes, many did not realize that cigarette smoking was a causative risk factor for type 2 diabetes (24). Our results reinforce the importance of knowledge and education with respect to smoking cessation. We did not see the significant changes in cigarette smoking among adults with diabetes in the young or older age groups, but this could be partially explained by more yearly type 2 diabetes incidence among middle-aged adults (25).

More than 1 in 3 young and middle-aged adults with 2 or more chronic diseases report current cigarette smoking. People who smoke cigarettes and have multiple chronic diseases appear to seek health care services more frequently and are more likely to try to quit with the support of evidence-based cessation treatments such as nicotine replacement therapy (26), yet the increased number of quit attempts and use of evidence-based cessation methods did not appear to equate to increased smoking cessation success (26). Viewing cigarette smoking as a chronic disease and, therefore, using chronic disease management methods for smoking cessation might help adults achieve smoking cessation (26). The use of these methods has been associated with both short-term and long-term smoking cessation versus usual care (27).

Young and middle-aged adults with chronic disease consistently had a prevalence of current cigarette smoking that was higher than the prevalence among older adults. In many cases, estimates were more than double. These findings have several possible explanations. Overall, cigarette smoking prevalence tends to be higher among young age groups, regardless of chronic disease status (2). Another possible explanation is a lower prevalence of traditional risk factors (eg, hypertension, hyperlipidemia) for chronic disease among young populations that typically lead to the development of chronic disease in older populations (20). Therefore, because of the low prevalence of these risk factors among young populations, smoking is more likely to be a primary risk factor for chronic disease in young populations (20). Additionally, the health effects of smoking are cumulative. Therefore, right censoring caused by increased likelihood of overall mortality among older populations may be a contributing factor in these findings. Additionally, age disparities in cigarette smoking may result from fewer visits to health care professionals, lack of tobacco use assessments, or low levels of tobacco cessation counseling among young adults who smoke cigarettes (18). The prevalence of tobacco counseling during outpatient visits has been previously reported as 14.5% among adults aged 18 to 24 years, compared with 22.1% among adults aged 45 to 64 years (18). Frequency of cessation advice provided by health care professionals has increased since 2000 (18). Yet almost 1 in 3 adults who smoke and have a chronic disease associated with smoking are not receiving advice to quit during their annual health care visits (18). Further research examining cessation rates among adults with chronic disease may further contextualize the findings of this report.

Approximately 1 in 4 young and middle-aged adults with COPD, CHD, stroke, diabetes, cancer associated with smoking, or people with 2 or more of these chronic diseases report current cigarette smoking. Smoking cessation can reduce morbidity and mortality risk in these populations. Using evidence-based cessation treatments, health care professionals can support the estimated 72.7% of adults aged 25 to 44 years and 68.7% of adults aged 45 to 64 years who report an interest in quitting (9,28). By quitting smoking, individuals with CHD can reduce their overall risk of mortality and risk of a new cardiac event, and disease and symptom progression of COPD can be slowed (18). Cancer survivors can improve their overall prognosis and might have the potential to decrease their mortality risk by quitting smoking (1,18,29). The results of this study indicate a need to provide appropriate smoking cessation services at the right time and in the right setting to adults living with chronic diseases. In addition, public health can help work toward reducing smoking among adults with chronic disease by continuing outreach of representative campaigns, such as Tips from Former Smokers, a Centers for Disease Control and Prevention media campaign that has frequently included people with cancer, COPD, CHD, and diabetes.

### Limitations

Several limitations apply to this study. First, cigarette smoking status and health outcomes were self-reported, resulting in potential recall and social desirability bias. Second, temporality of smoking initiation among those who currently smoke and quitting among those who formerly smoked cigarettes is unknown. Third, we were unable to distinguish type 1 and type 2 diabetes in the survey and could look at only those chronic diseases assessed in NHIS (eg, reason for exclusion of acute myeloid leukemia). Fourth, NHIS underwent changes to the nonresponse adjustment to sample weighting and a questionnaire redesign in 2019 (11), so comparisons using 2019 data must be interpreted with caution. Lastly, while this study provides evidence of an opportunity to improve clinical cessation services among adults with chronic disease, smoking is not always captured in clinical data (ie, electronic health records) and, therefore, might lead to a missed opportunity to provide cessation services. Even if smoking information is captured in clinical data, that information is not always used.

### Conclusions

Our study provides updated estimates of current and former cigarette smoking among adults aged 18 years or older with chronic diseases associated with cigarette smoking. This study also provides new information on cigarette smoking trends among adults with chronic diseases over a 10-year period. Only one significant decrease in cigarette smoking was reported among age groups with chronic diseases over the past 10 years (middle-aged adults with diabetes), relative to the overall decrease in smoking prevalence seen among all US adults (2). The results of this study indicate a consistent prevalence of cigarette smoking and a lack of progress over time in smoking reduction in these populations, who, in addition, are at risk of further complications by continuing to smoke. Cessation advice and services are not being provided to almost 1 in 3 people who have a chronic disease (18). Greater access to cessation services, integration of cessation treatment into routine care in all clinical settings, recognition that people who smoke might benefit from a chronic disease–type management model, and long-term follow up and support may be important steps to take toward successful smoking cessation in this population (29).
